# Editorial: Overcoming drug relapse and therapy resistance in NSCLC

**DOI:** 10.3389/fonc.2023.1230475

**Published:** 2023-06-07

**Authors:** Sagun Parakh, Tracy L. Leong, Sarah A. Best, Ashleigh R. Poh

**Affiliations:** ^1^ Department of Medical Oncology, Austin Health, Heidelberg, VIC, Australia; ^2^ Tumor Targeting Laboratory, The Olivia Newton-John Cancer Research Institute, Heidelberg, VIC, Australia; ^3^ School of Cancer Medicine, La Trobe University, Melbourne, VIC, Australia; ^4^ Department of Respiratory Medicine, Austin Health, Heidelberg, VIC, Australia; ^5^ Faculty of Medicine, University of Melbourne, Parkville, VIC, Australia; ^6^ Personalised Oncology Division, The Walter and Eliza Hall Institute of Medical Research, Parkville, VIC, Australia; ^7^ Department of Medical Biology, University of Melbourne, Melbourne, Australia; ^8^ Cancer and Inflammation Laboratory, The Olivia Newton-John Cancer Research Institute, Heidelberg, VIC, Australia

**Keywords:** drug resistance, non-small cell lung cancer, metabolism, tumor microenvironment, biomarkers, tumor mutational burden (TMB), EGFR mutation, immunophenotyping

Non-small cell lung cancer (NSCLC) is the most common type of lung cancer with a 5-year survival rate of less than 25% (1). Current treatment modalities include surgery, radiotherapy, and chemotherapy; however, most individuals are diagnosed at a locally advanced or metastatic stage when curative treatments are unavailable (2). Despite the success of targeted therapies and immunotherapies, drug resistance continues to be a major challenge in the management of NSCLC. Tumor cells can develop drug resistance through several mechanisms, including the acquisition of genetic mutations, aberrant activation of signaling pathways, alterations in the tumor microenvironment, and selection for drug-resistant subpopulations (3). As a result, patients often experience disease progression and relapse after initial response to therapy. In some cases, complete responders with minimal to no detectable disease post-treatment eventually develop drug resistance and succumb to tumor progression.

This Research Topic focuses on the latest research findings and insights into the immunological and molecular mechanisms that underpin NSCLC drug relapse and therapy resistance. The articles in this Research Topic cover a range of topics, including the identification of novel biomarkers to predict resistance, tumor heterogeneity in metastatic disease, metabolic vulnerabilities, and the design of new strategies to improve treatment outcomes. Together, they provide a comprehensive overview of the current state of research into NSCLC drug resistance, and highlight promising new directions for future studies.

Accurate assessment of the tumor microenvironment is essential to understanding mechanisms of treatment resistance. Reviewed in this Research Topic, Rangamuwa et al. describe “immune phenotyping” which characterizes the immune milieu of the tumor, and may provide insights into response to immune-modulating therapy. A range of techniques may be employed to examine the tumor microenvironment, including immunohistochemistry, cytometry, and transcriptomic analysis. The utility of such modalities, particularly with small-volume tissue samples obtained from patients with advanced stage disease, is an area of growing interest (4).

Identifying predictive and prognostic biomarkers has emerged as a promising strategy for overcoming drug resistance and improving treatment outcomes by enabling more targeted and personalized approaches to patient care. In an original research article, Zhang et al. identified elevated expression of the cell cycle regulatory protein CDC25C as an independent prognostic biomarker for poorer overall survival and disease progression in lung adenocarcinoma patients. Notably, increased *CDC25C* expression also correlated with an immunosuppressive tumor microenvironment and reduced response to anti-PD1 therapy. The dual role of CDC25C as both a prognostic and predictive biomarker has potential to improve patient outcomes by guiding treatment decisions and identifying those who are likely to benefit from immune checkpoint blockade.

Tumor mutational burden (TMB) is recognised as potential biomarker of responsiveness to immunotherapy. While there is evidence of genomic heterogeneity between tumor regions in the individual patient (5), there is less understanding of whether similar heterogeneity exists when deriving the TMB. In an original study by Leong et al., TMB was assessed in a cohort of patients with paired primary and metastatic tumor samples. Whole exome sequencing and targeted cancer-specific sequencing showed relative stability of TMB values across distinct tumor regions. Further work, however, is required to improve precision of the TMB threshold of clinical relevance.

Minimal residual disease in NSCLC is defined as micro-metastatic disease that persists after initial therapy, representing a potential source of subsequent metastatic relapse at distant sites (6). In the accompanying editorial, Bain et al. discuss strategies to target minimal residual disease in *EGFR*-mutant NSCLC by targeting senescence-like cell dormancy and enhancing apoptosis by combining EGFR TKIs with other therapeutic agents. These strategies are aimed at achieving deeper responses and potentially preventing/delaying drug resistance. However, biomarkers that can accurately detect minimal residual disease in solid tumors to identify patients at risk of relapse, escalate or de-escalate treatment, and determine optimal treatment duration remains elusive.

In the metastatic setting, the third-generation tyrosine kinase inhibitor, osimertinib has been approved in NSCLC with common EGFR mutations (L858R or Exon 19 deletions). Despite striking benefits, resistance invariably occurs against osimertinib and the efficacy of EGFR-TKIs in uncommon *EGFR* mutations (a highly heterogeneous group of molecular alterations within exons 18 to 21) is variable. Effective strategies to target these alterations is a clinically unmet need. Zhang et al. reviewed the potential of using EGFR-targeting monoclonal antibodies in combination with TKIs to treat this patient population. This strategy has shown to produce synergistic antitumor effects albeit high toxicity rates. Novel therapies such as the EGFR–MET-targeted bispecific antibody, amivantimab, and tyrosine kinase inhibitors that specifically target uncommon *EGFR* mutations (e.g., mobicertinib), have shown promise and continue to be evaluated in this patient population in combination with other therapies.

Altered metabolic properties of cancer cells can also play a driving role in drug relapse and therapy resistance. By exploiting metabolic pathways to increase antioxidant production to bolster cellular protection and harness glucose-independent energy production using glutamine, cancer cells can become dependent on this non-essential amino acid. Reviewed in this Research Topic by Tang et al., the therapeutic potential of glutamine metabolism has been explored in lung cancer. In addition to anti-cancer benefits of targeting the glutamine pathway, this review additionally highlights that glutamine supplementation during radiation therapy could mitigate the severity of complications during radiotherapy for lung cancer patients.

In response to radiation and alkylating agents in lung cancer, Chang et al. found that an Aldolase A (ALDOA) and Phospholipase D1 (PLD1) axis drives a more aggressive phenotype and may represent a target for inhibition. The ALDOA/PLD1 axis is crucial for enhancing the proliferation of lung cancer cells, autophagy, and DNA repair capabilities after exposure to alkylating agents and radiation. The study also found that ALDOA can inhibit the activity and enzyme function of PLD2 through direct protein-protein interaction to enhance the activity of PLD1 and additional carcinogenic features. Together, these two papers provide insights into the metabolic vulnerabilities in lung cancer cells and the potential of targeting specific metabolic pathways as a personalized therapy approach.

Collectively, the articles in this Research Topic provide an important snapshot of the current challenges and ongoing efforts to understand the mechanisms of drug relapse and therapy resistance in NSCLC. Additional studies that build on these findings will be critical to advance the development of more effective treatment strategies to improve patient outcomes and survival.

## Author contributions

All authors listed have made a substantial, direct, and intellectual contribution to the work and approved it for publication.
